# The influence of glacial melt and retreat on the nutritional condition of the bivalve *Nuculana inaequisculpta* (Protobranchia: Nuculanidae) in the West Antarctic Peninsula

**DOI:** 10.1371/journal.pone.0233513

**Published:** 2020-05-21

**Authors:** Miguel Bascur, Carlos Muñoz-Ramírez, Alejandro Román-González, Katy Sheen, David K. A. Barnes, Chester J. Sands, Antonio Brante, Ángel Urzúa

**Affiliations:** 1 Departamento de Ecología, Facultad de Ciencias, Universidad Católica de la Santísima Concepción, Concepción, Chile; 2 Programa de Magíster en Ecología Marina, Universidad Católica de la Santísima Concepción, Concepción, Chile; 3 Centro de Investigación en Biodiversidad y Ambientes Sustentables (CIBAS), Universidad Católica de la Santísima Concepción, Concepción, Chile; 4 Instituto de Entomología, Universidad Metropolitana de Ciencias de la Educación, Santiago, Chile; 5 College of Life and Environmental Sciences, University of Exeter, Penryn, Cornwall, England, United Kingdom; 6 British Antarctic Survey, Natural Environment Research Council, Cambridge, England, United Kingdom; Union College, UNITED STATES

## Abstract

Due to climate change, numerous ice bodies have been lost in the West Antarctic Peninsula (WAP). As a consequence, deglaciation is expected to impact the marine environment and its biota at physiological and ecosystem levels. *Nuculana inaequisculpta* is a marine bivalve widely distributed around Antarctica that plays an important role for ecosystem functioning. Considering that *N*. *inaequisculpta* inhabits coastal areas under effect of glacial melt and retreat, impacts on its nutritional condition are expected due to alterations on its physiology and food availability. To test this hypothesis, biochemical composition (lipids, proteins, and fatty acids) and energy content were measured in individuals of *N*. *inaequisculpta* collected in a fjord at different distances to the retreating glacier in the WAP. Oceanographic parameters of the top and bottom-water layers (temperature, salinity, dissolved oxygen, and chlorophyll-a) were measured to investigate how the environment changes along the fjord. Results showed that surface oceanographic parameters displayed a lower temperature and dissolved oxygen, but a higher salinity and chlorophyll-a content at nearest compared to farthest sites to the glacier. In contrast, a lower temperature and chlorophyll-a, and a higher salinity and dissolved oxygen was measured in the bottom-water layer toward the glacier. *N*. *inaequisculpta* had a higher amount of lipids (17.42 ± 3.24 vs. 12.16 ± 3.46%), protein (24.34 ± 6.12 vs. 21.05 ± 2.46%) and energy content (50.57 ± 6.97 J vs. 39.14 ± 5.80 J) in the farthest compared to the nearest site to the glacier. No differences were found in total fatty acids among all sites. It seems likely that lower individual fitness related to proximity to the glacier would not be related to nutritional quality of sediment food, but rather to food quantity.

## Introduction

The Antarctic marine environment has long been one of the most stable environments in the world due to its marked seasonality, especially regarding ice dynamic and availability of planktonic food [1,2], but also one of the most vulnerable to global warming [3,4]. Early 20^th^ century measurements found a rapid increase in air temperature, reaching an increase of +5.6°C over the century average temperature [5]. Currently, one of the most impacted areas in Antarctica is the Western Antarctic Peninsula (WAP) due to warming of the Circumpolar Deep Water (CDW), produced by barotropic Kelvin waves [6], which have a direct influence on certain parts of the Antarctic continental shelf [7]. Hence, warming shelf has led to constant losses of sea ice and the melting/retreat of glaciers in small fjords along the WAP [7,8,9].

Temporal and spatial variations have been registered in the amount of food available in Antarctic sediment, making Antarctic marine invertebrates that feed on deposited organic matter vulnerable [10,11]. On the one hand, recent studies found seasonal changes in the amount of food available in the sediment, with a higher amount of lipids and proteins (high quality food) during the autumn, due to the vertical exportation of the last summer phytoplankton blooms and a higher amount of carbohydrates (low food quality) during spring [11]. On the other hand, spatial changes have been observed in the amount of food available in the sediment in Antarctic places with an ancient and recent loss of ice shelves [10]. The results show that there is a higher amount of lipids and proteins and a lower amount of carbohydrates in the sites with an ancient loss than in the sites with a recent loss of the ice shelf [10]. In this context, spatial changes in the amount of food available in the sediment could be expected due to the recent melting and retreat of glaciers in small WAP fjords, which have made available new ice-free habitats [12]. However, the effect of glacier melting and retreat on the energetic storage and fitness of benthic species in the WAP is still poorly understood.

From a physiological point of view, the fitness of an organism is the ability to respond efficiently to environmental or biological variations through physiological and biochemical mechanisms [13,14]. The fitness responds to adjustments of the energy budget made by the organism and consequently, an individual can only store energy when the absorption of nutrients exceeds the energy budget demand (i.e. under high food availability). Therefore, a greater amount of stored energy would indicate greater fitness (i.e. higher survival and reproduction rates), especially under stressful environments [13]. In this context, the glacier melting and retreat can modify some oceanographic variables (e.g. temperature and salinity) and food availability in sediments [15,16,17], and thus could have significant impacts on the physiology and energy storage of benthic species. In this way, measuring nutritional condition of benthic species can be used as a proxy to evaluate the impact of glacial melting and retreat on food availability in sediment of Antarctic marine ecosystems.

Fitness is measurable through indicators of “nutritional condition” and have been commonly studied by means of biochemical components storage in marine bivalves of different regions: tropical (e.g. *Lyropecten nodosus*) [18], temperate (e.g. *Mytilus edulis*, *Mytilus galloprovinvialis*, *Crassostrea gigas*) [19], subpolar (e.g. *Yoldia hyperborea*) [20] and polar regions (e.g. *Laternula elliptica*) [21]. Additionally, within biochemical components, lipid and protein represent the higher energetic values and they play an essential role on bivalve’s reproduction success [22]. The biochemical composition has also crucial functions during development of early stages, since it has been described that dry weight of several bivalve eggs are composed by 40–50% of protein and 14–25% of lipid [23,24,25,26]. Thus, any variation in the biochemical composition of adult individuals could have direct effects on the reproductive energy investment and viability of the offspring produced.

*Nuculana inaequisculpta* (Lamy 1906) is a small size bivalve mollusc species that belongs to the Protobranchia subclass, a taxonomic group that contains the most ancient bivalve molluscs in the world [27,28]. It is an abundant infaunal species, patchy distributed in the South Shetland Islands and Antarctic Peninsula [29,30,31]. This species lives in benthic, muddy marine habitats and feed mainly on sediment with organic deposits [32]. Documented individuals have been captured at maximum depths ca. 800 m with size between 2.5–16 mm of shell length [29,31]. Additional characteristics on the biology and ecology of this particular species are common within the Protobranchia subclass. Species belonging this subclass have two separate sexes, with a similar sex ratio (ca. one male for each female), high fecundity, and a lecithotrophic larval development with a pericalima larva [32]. Due to the particular important role as nutrient recyclers and its high abundance [29,30,31], the present study focused on *N*. *inaequisculpta* to assess whether glacial melting and retreat, a product of regional warming, has an effect on the nutritional status (i.e. fitness) of benthic species that inhabit Antarctic fjords. Additionally, the potential implications of the nutritional status of the species on the stability of its population on the Antarctic food web is also discussed.

## Methods

### Collection of oceanographic parameters

**Fig 1:**
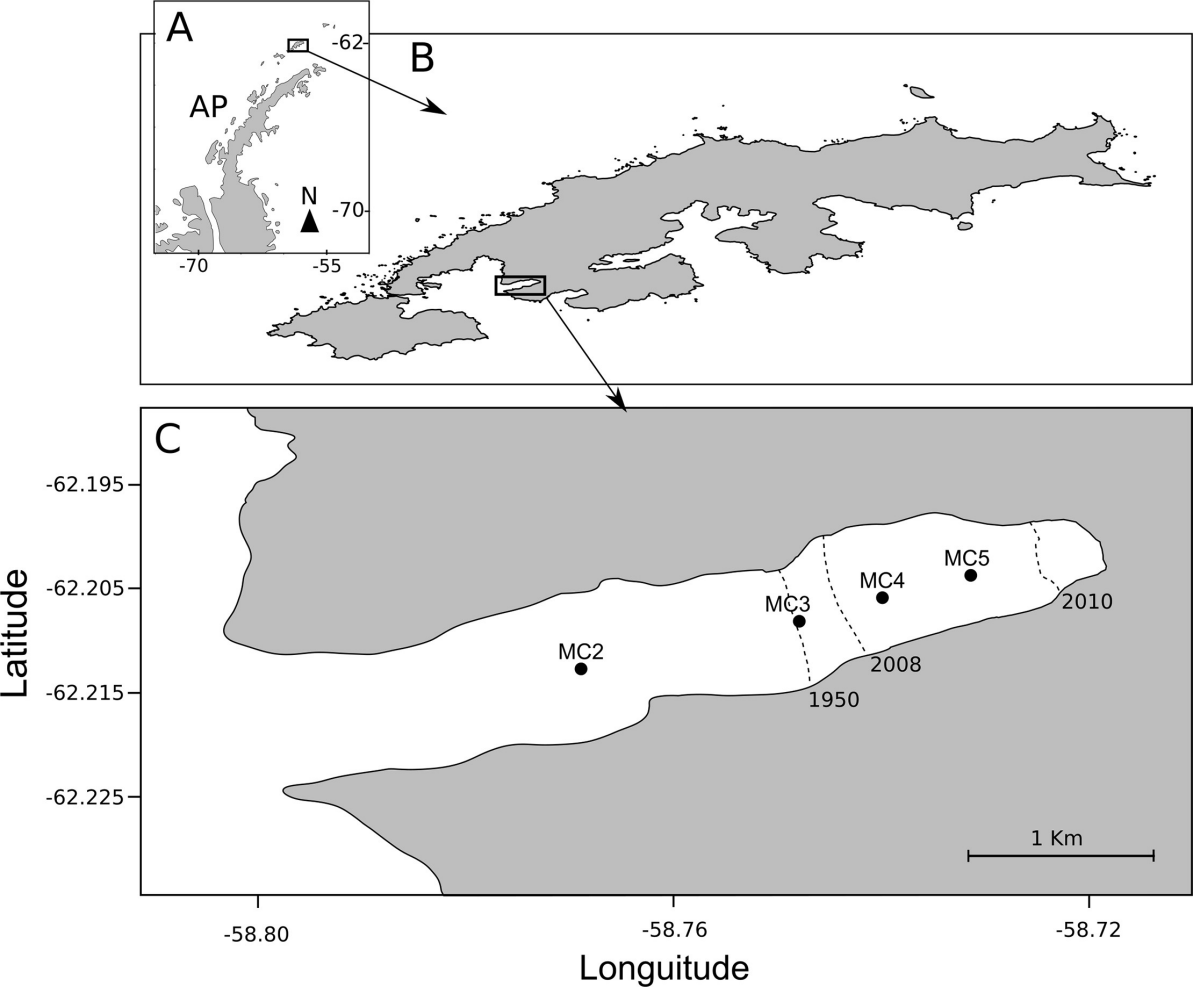
Map showing the geographic location of the Antarctic Peninsula **(A)**, King George Island **(B)** and Marian Cove **(C)**. The white filled circles indicate the sampling sites. Dashed lines indicate the historical record of the glacier margin within the fjord in different years.

Marian Cove (MC) is an inlet within Maxwell Bay, located in the southeast of King George Island (South Shetland Islands, WAP, Fig 1). The marine terminating glacier system at this location has experienced a significant retreat in recent decades [15,33]. Onboard the RRS James Clark Ross vessel, oceanographic measurements from the CONICYT-NERC project campaign "ICEBERGS 1", in November 2017, were taken from four sites along Marian Cove from the inlet opening to the proximity of the glacier margin. The sites were classified as follows: MC2 has not had contact with the glacier margin since the last glacial maximum. MC3 corresponds to the place where the glacier was located in the year 1950, while MC4 is located near the glacier margin in 2008. Finally, MC5 is near the glacier margin in 2010 (Fig 1).

In order to characterize the system, measurements of seawater temperature, salinity, dissolved oxygen and chlorophyll-a (fluorescence) were obtained during November 2017, through a vertical profile of the water column with the SeaBird 911plus CTD (Conductivity-Temperature-Depth) system onboard the RRS James Clark Ross (JCR, British Antarctic Survey). Rosettes with Niskin bottles were used to collect discrete water samples for salinity analysis. Then, to verify the salinity of the CTD measurements, the discrete samples were analyzed with Autosal 8400B. Dissolved oxygen data (μmol/kg) was transformed to mg/L using the molar mass of oxygen and density of seawater based on salinity and temperature data recorded. Dissolved oxygen data in both measurement units (μmol/kg and mg/L) are available in supplementary information (S2 File).

Top-water layer oceanographic parameters were studied independently from bottom-water layer parameters. The bottom-water layer oceanographic parameters were separated to assess whether a differential effect on the physiology of *N*. *inaequisculpta* could be detected along the transect. Therefore, from the full vertical profile, data from the first 10 m (15 replicates per study site and per oceanographic variable) and the last 10 m depth (15 replicates per study site and per oceanographic variable) were selected and grouped to perform analyses. However, given that the vertical profile of the site nearest to the glacier (MC5) only measured up to 70 m depth but the seafloor was at a greater depth (over 100 m deep), we excluded this site from the analyses.

### Nutritional condition of *N*. *inaequisculpta*

#### Sample collection

To assess the effect of deglaciation on the nutritional condition of *N*. *inaequisculpta*, large specimens (> 8 mm of shell length) were collected close to the sites where oceanographic data were measured (S1 Table, Fig 1). A total of 160 individuals (40 individuals per site) were extracted using a Van Veen grab with a 20 cm x 40 cm x 40 cm bucket at a water depth between 100 and 115 m. The samples were preserved in 99% ethanol and frozen at -80°C. Then, samples were transported to the Hydrobiological Resources Laboratory of the Universidad Católica de la Santísima Concepción and stored in the same conditions until the analysis. Last, of the 160 individuals collected, two groups of 80 individuals each were separated to proximal biochemical composition analysis: 1) lipid content and fatty acids, 2) protein content.

#### Size and biomass

In the laboratory, shell length, defined as the distance between the anterior and posterior edge of the shell, was measured in 160 individuals using a Vernier caliper (0.01 mm precision). To determine the body mass without shell, salt and sediments were removed from the soft tissue of each individual using abundant distilled water on a 0.2 mm sieve. Clean samples were stored in labeled Eppendorf tubes, and then were frozen at -20°C, and dried by sublimation in a lyophilizer (FDU-7012, Operon) for 48 h at -80°C. Once dried, biomass of 160 individuals, defined as the dry weight of the individuals, was determined using an analytical balance of 0.1 mg sensitivity (LA230S SARTORIUS).

#### Proximate biochemical composition (lipid and protein content)

The biochemical composition (i.e. total lipid and protein content) was determined in 4 mg of homogenized dry weight (DW) for each individual and expressed in absolute (mg * 4mg^–1^) and relative (% dry weight, DW) values. To improve the performance of the biochemical tests, the samples were subjected to an ultrasonic bath (AC-120H, MRC) with distilled water, for 15 minutes at 6°C, prior to the following analyses.

The total lipid content was determined in 80 dry and ethanol-preserved samples (20 individuals per study site) following the gravimetric method set by Folch et al. [34] and later modified by Cequier-Sánchez et al. [35]. First, each dried sample was homogenized in labeled amber tubes with 5 mL of dichloromethane: methanol mix (2:1). Then, the samples were mixed with 4 mL 0.88% potassium chloride, mixed by vortex (SBS100-2, Select Vortexer) for 15 seconds, and centrifuged (S-8, Boeco) for 5 minutes at 6°C and 1500 rpm. Subsequently, the precipitate of each sample was transferred to pre-weighed vials and dried with ultrapure nitrogen gas to evaporate the solvent (109A YH-1, Glass Col). The total lipid extract obtained after evaporating the solvent was calculated by weighing the full vial (containing the lipid extract) on a precision balance (120A, Precise) and subtracting the empty vial’s weight. Finally, the lipid extract of each sample was preserved at -80°C in dichloromethane: methanol mix (2:1) with Butylhydroxytoluene (BHT) as antioxidant to avoid the degradation of fatty acids for future analysis.

The total protein content was determined in the remaining 80 samples (20 individuals per study site) through a microplate adaptation of the BIO-RAD colorimetric assay kit, by Lowry et al. [36], which includes three reagents: S, A, and B. Therefore, 4 mg of dry weight per individual was homogenized in 200 μL of ultrapure water (Mili-Q), then 5 μL of the homogenized suspension was transferred to a 96-well microplate with 200 μL of Reagent B and 25 μL of Reagent A' (i.e. a mixture of 20 μL of Reagent S and 1 mL of Reagent A). Subsequently, the samples were shaken for 15 seconds by vortex (SBS100-2, Select Vortexer) and incubated in the microplates for 15 minutes at room temperature. Finally, the absorbance was measured in a spectrophotometer at a wavelength of 750 nm (ELx808, Biotek). The concentration of each sample was obtained using a calibration curve for proteins, diluting different concentrations of bovine serum albumin (500–0111, Bio-Rad).

#### Energy content

The energy content (J * 4mg^–1^) of 80 individuals was estimated from the biochemical composition data (i.e. lipid and protein contents) using bioenergetic equivalents. The bioenergetic equivalents were calculated using the following conversion coefficients: (a) 1 mg of lipids = 39.54 J and (b) 1 mg of protein = 23.64 J [37]. An approximation of the total energy content for each individual was calculated by adding both energy equivalents of the biochemical composition (as Total Energy = J * mg lipids + J * mg proteins) [37,38,39]. Carbohydrates are not considered due their minor contribution to the sample’s total biomass.

#### Fatty acids composition

The composition of fatty acids was determined in 80 samples (20 individuals per study site) using standard methods [40,41]. Fatty acid methyl esters (FAMEs) were measured after preparation using the lipid extract of samples (lipid content). Lipid extracts were esterified using sulfuric acid (1% in methanol) incubations at 70°C for 1 h in a Thermo-Shaker (DBS-001, MRC). Then, each sample was mixed with 3 mL of n-hexane and centrifuged for 15 s. This process was repeated three times and the supernatant was transferred to labeled tubes. Finally, fatty acids were concentrated using a nitrogen evaporator (109A YH-1, Glass Col). The measurement of FAMEs was performed using a gas chromatograph (Agilent, model 7890A) at set temperature equipped with a DB-225 column (J&W Scientific, 30 m in length, 0.25 internal diameter, and 0.25 mm film). Using chromatograph software (Agilent ChemStation, USA), individual FAMEs were identified by comparison to known standard fatty acids of marine origin (certificate material, Supelco 37 FAME mix 47885-U [40,42]. Each sample was quantified using a calibration curve for fatty acids, diluting different concentrations of Supelco 37 FAME mix standard.

### Statistical analysis

All the statistical analyses were performed in STATISTICA V8 and PRIMER V6 (+ PERMANOVA), with a 95% confidence level (*p* < 0.05), based on standard methods [43,44,45]. Each top and bottom-water layer oceanographic parameter data obtained in all study sites (i.e. temperature, salinity, dissolved oxygen, and chlorophyll-a) were analyzed with a nonparametric Kruskal-Wallis test. The size, biomass and protein content of *N*. *inaequisculpta* individuals obtained in the different study sites were evaluated by a Kruskal-Wallis test, while the other nutritional parameters of individuals (i.e. lipid content, energy, and fatty acids) were evaluated using one-way ANOVA test. For both, oceanographic and nutritional variables, analyses were performed with the “site” factor [with four levels: MC2 (farthest to the glacier), MC3, MC4 and MC5 (nearest to the glacier)]. All results are shown as mean values, with standard deviation (±SD). The assumptions of ANOVA were evaluated with the Kolmogorov-Smirnov and the Levene’s tests for the normality and homogeneity of variances, respectively. When significant statistical differences were found for the ANOVA or Kruskal-Wallis test, a Tukey test or a multiple range test with a Bonferroni correction was performed to estimate differences between treatments.

Additionally, multivariate analyses were carried out in PRIMER V6 to compare the composition of fatty acids. First, a one-way PERMANOVA analysis was performed to evaluate the complete data set of fatty acids. Then, to evaluate the percentage of contribution of each fatty acid to similarity between treatments, a similarity percentage analysis (SIMPER) was carried out.

## Results

### Oceanographic parameters

#### Sea surface parameters (top 10 m)

**Fig 2:**
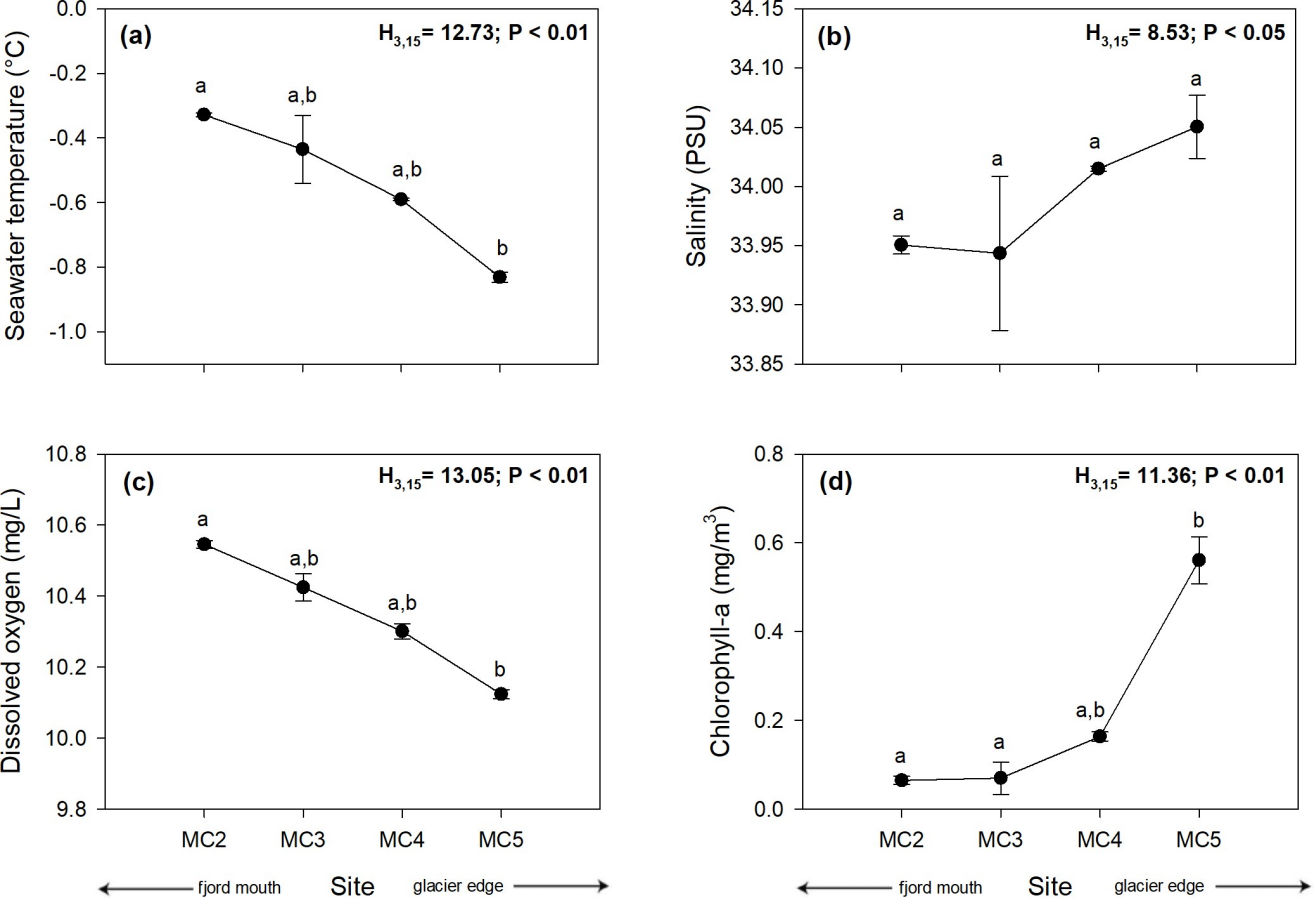
Oceanographic instrumental variability for the top 10 m of the seawater column. Plot **(a)** temperature (°C), **(b)** salinity (PSU), **(c)** dissolved oxygen (mg/L), and **(d)** chlorophyll-a (mg/m^3^). The four sites are at different distances from a melting glacier (MC2 distal, MC5 proximal) in Marian Cove, WAP. Different letters on black circles indicate significant differences among sites after multiple range test with a Bonferroni correction. Measurements are given as average values ± SD (*n* = 15).

Sea surface temperature showed significant differences among sites (Kruskal-Wallis test, H_3,15_ = 12.73; *p* < 0.001) with a clear pattern of declining temperature towards the glacier edge (MC5). The highest temperature was recorded in MC2 (–0.33 ± 0.006°C), followed by MC3 (–0.44 ± 0.11°C), and MC4 (–0.59 ± 0.004°C); while the lowest temperature was recorded in MC5 (–0.83 ± 0.016°C) (Fig 2A; S2 Table). Similar to the temperature results, salinity had significant differences among study sites (Kruskal-Wallis test, H_3,15_ = 8.53; *p* < 0.05). Lower surface salinity was found at the farthest (MC2: 33.95 ± 0.008 PSU; MC3: 33.94 ± 0.7 PSU) compared to the nearest sites to the glacier (MC4: 34.02 ± 0.002 PSU; MC5: 34.05 ± 0.03 PSU) (Fig 2B, S2 Table).

Dissolved oxygen also showed significant differences among the study sites (Kruskal-Wallis test, H_3,15_ = 13.05; *p* < 0.01) and declining toward the glacier. The highest amount of dissolved oxygen was found at the farthest site from the glacier (MC2: 10.55 ± 0.01 mg/L), followed by the MC3 site (10.42 ± 0.04 mg/L), MC4 (10.30 ± 0.02 mg/L) and MC5 (10.12 ± 0.01 mg/L) (Fig 2C, S2 Table). The amount of surface chlorophyll-a also showed significant differences (Kruskal-Wallis test, H_3,15_ = 11.36; *p* < 0.01), with a lower amount of chlorophyll-a at the more distant sites (MC2: 0.065 ± 0.01 mg/m^3^; MC3: 0.070 ± 0.03 mg/m^3^) and higher amounts at sites near the glacier front (MC4: 0.16 ± 0.01 mg/m^3^; MC5: 0.56 ± 0.05 mg/m^3^) (Fig 2D, S2 Table).

#### Sea depth parameters (bottom 10 m)

**Fig 3:**
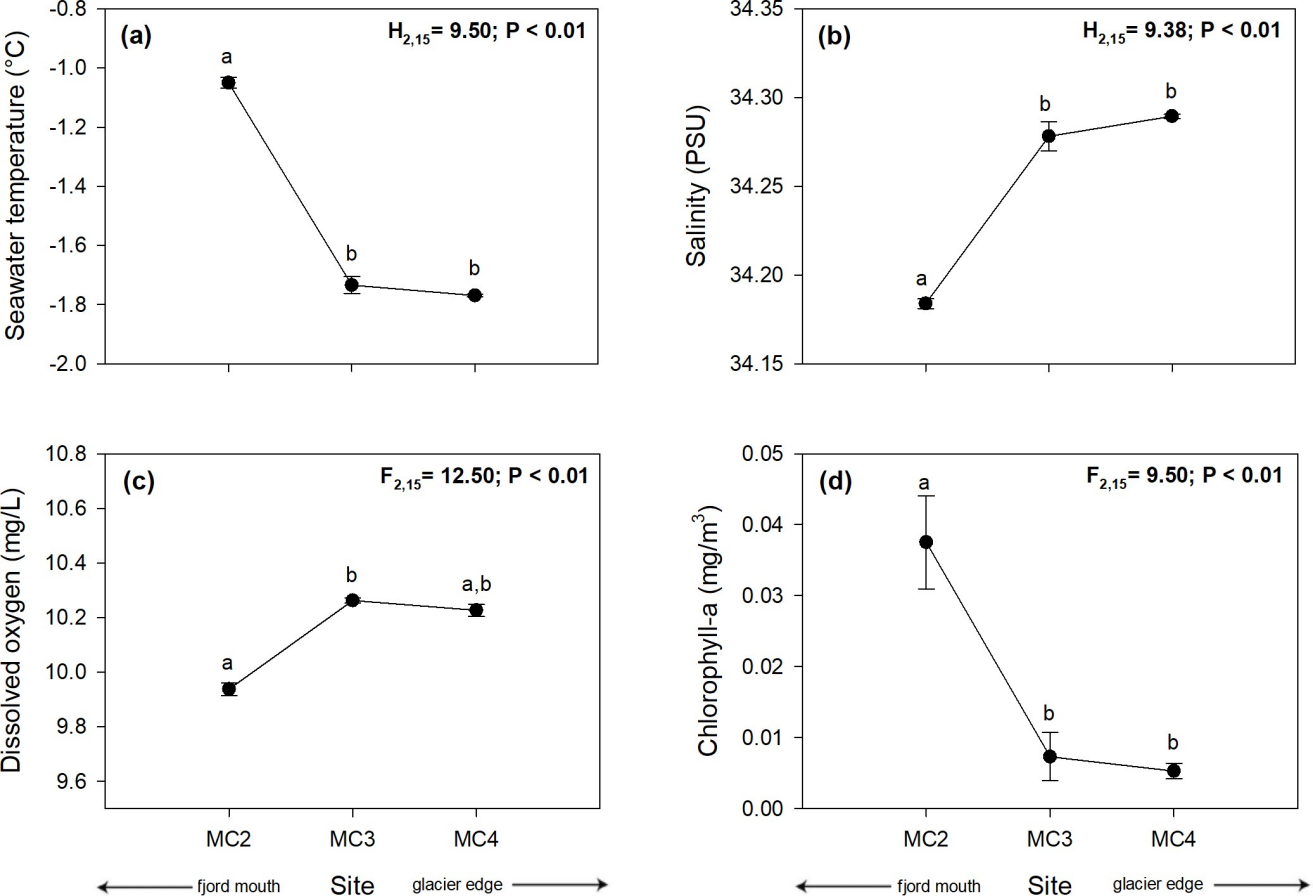
Oceanographic instrumental variability for the bottom 10 m of the seawater column. Plot **(a)** temperature (°C), **(b)** salinity (PSU), **(c)** dissolved oxygen (mg/L), and **(d)** chlorophyll-a (mg/m^3^). The four sites are at different distances from a melting glacier (MC2 distal, MC5 proximal) in Marian Cove, WAP. Different letters on black circles indicate significant differences among sites after multiple range test with a Bonferroni correction. Measurements are given as average values ± SD (*n* = 15).

The deep temperature profiles showed significant differences among the study sites (Kruskal-Wallis test, H_2,15_ = 9.5; *p* < 0.01) with a trend of decrease from distant to closer sites to the glacier. The highest temperature was recorded in MC2 (–1.05 ± 0.02°C), followed by MC3 (–1.73 ± 0.03°C), while the lowest was recorded in MC4 (–1.77 ± 0.004°C) (Fig 3A, S2 Table). Significant differences among salinities of the study sites (Kruskal-Wallis test, H_2,15_ = 9.38; *p* < 0,01) followed an opposite gradient, with the lowest salinity at the farthest site from the glacier (MC2: 34,18 ± 0,003 PSU), followed by MC3 (34.28 ± 0.008 PSU), and lastly MC4 with the highest values (34.29 ± 0.001 PSU) (Fig 3B, S2 Table).

Dissolved oxygen measurements were also significantly different among the study sites (Kruskal-Wallis test, H_2,15_ = 12.50; *p* < 0.01), with a decreasing trend similar to salinity. The lowest amount of dissolved oxygen was measured at the farthest site from the glacier (MC2: 9.94 ± 0.02 mg/L), while the highest was at the nearest sites to the glacier (MC3: 10.26 ± 0.01 mg/L; MC4: 10.23 ± 0.02 mg/L) (Fig 3C, S2 Table). In contrast to salinity and dissolved oxygen, the measurements of chlorophyll-a. showed the highest amount at the farthest site (MC2: 0.038 ± 0.007 mg/m^3^) than at the nearest sites to the glacier (MC3: 0.007 ± 0.003 mg/m^3^; MC4: 0.005 ± 0.001 mg/m^3^) (Fig 3D, S2 Table), with significant differences among all sites (Kruskal-Wallis test, H_2,15_ = 9.5; *p* <0.01).

### Nutritional condition of *N*. *inaequisculpta*

#### Size and biomass

**Fig 4:**
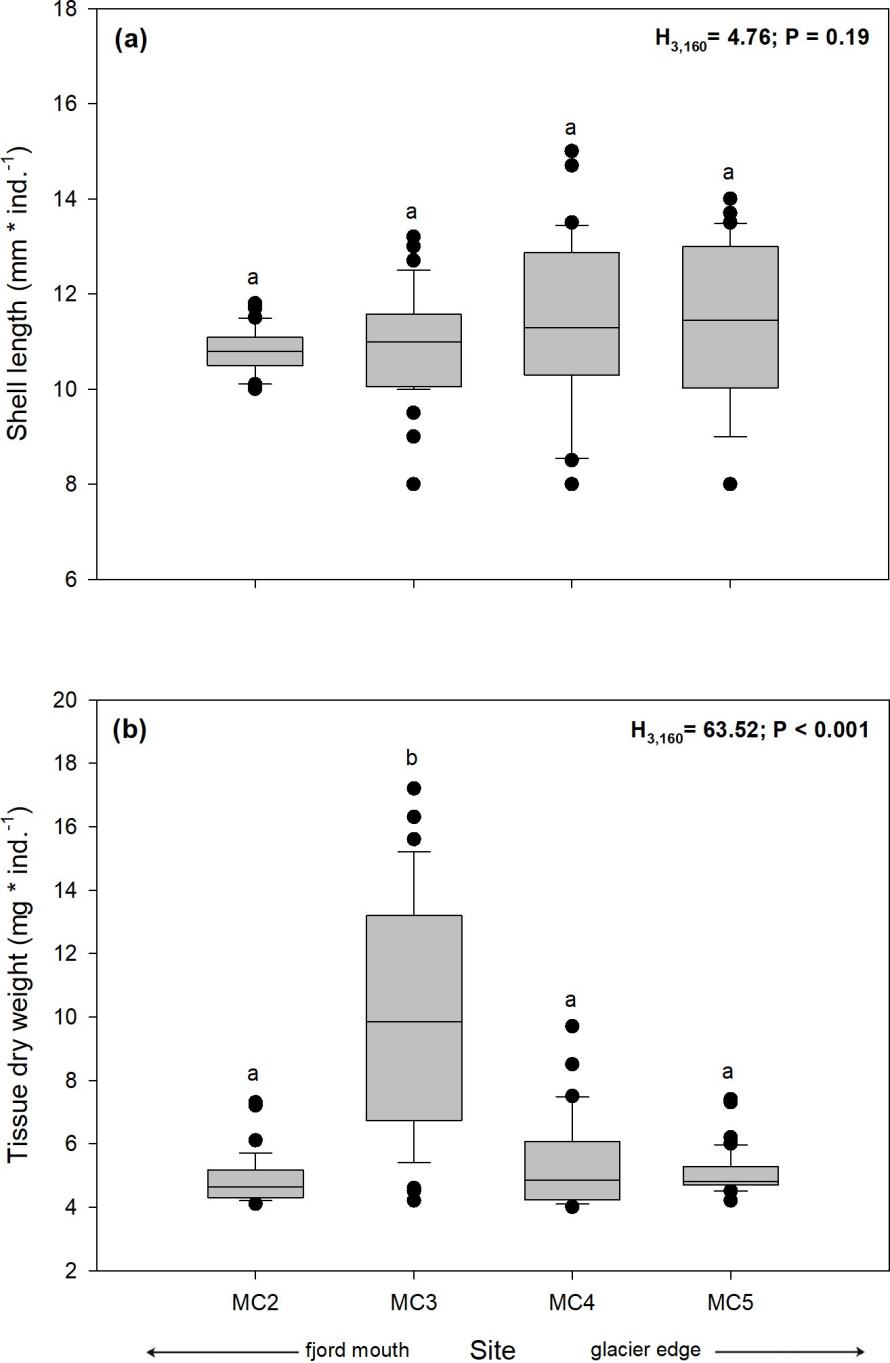
Boxplot of the spatial variation of: **(a)** shell length (mm * ind.^–1^) and **(b)** tissue dry weight (mg * ind.^–1^) of adult individuals of *N*. *inaequisculpta* caught at different four sites (MC2 to MC5) at progressive distance from a melting glacier (MC2 distal, MC5 proximal) in Marian Cove, WAP. Different letters on box indicate significant differences among sites after Tukey’s HSD test or multiple range test with a Bonferroni correction. Measurements are given as average values ± SD (*n* = 160).

No significant differences were found among sampling sites for individual shell length regardless of the distance to the glacier (Kruskal-Wallis test, H_3,160_ = 4.76; *p* = 0.19) (Fig 4A, S3 Table). Also, body biomass (as dry weight of soft tissue, mg * ind.^-1^) presented significant differences among sampling sites, where the individuals from the MC3 site showed a significantly higher body biomass (10.09 ± 3.73 mg) than those from MC2 (4.84 ± 0.75 mg), MC4 (5.30 ± 1.48 mg), and MC5 (5.07 ± 0.68 mg) (Kruskal-Wallis test, H_3,160_ = 63.51; *p* < 0.001) (Fig 4B, S3 Table).

#### Proximate biochemical composition (lipid and protein content)

**Fig 5:**
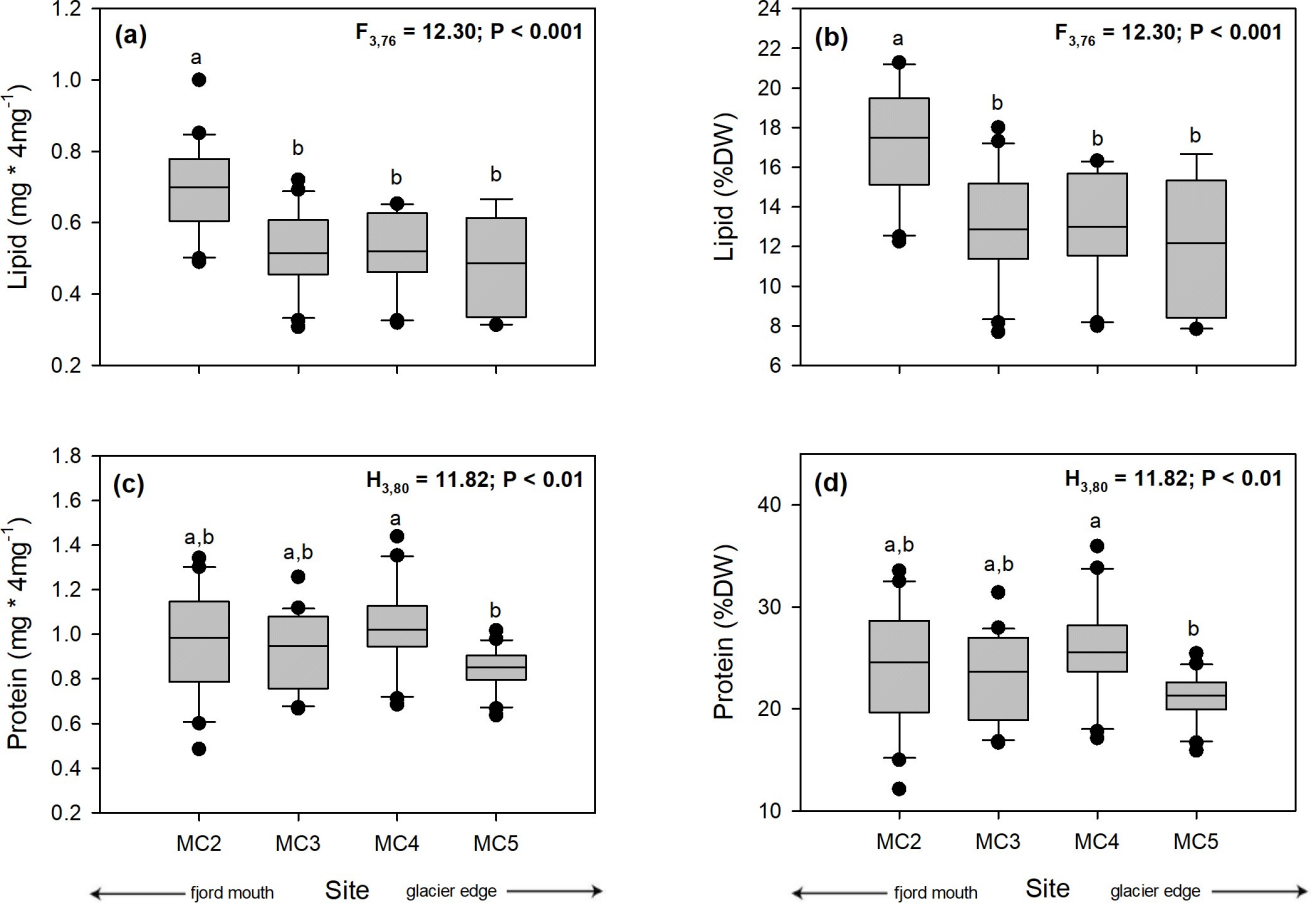
Boxplot of the spatial variation of: **(a)** lipid content (mg * 4mg^–1^), **(b)** lipid content (% DW), **(c)** protein content (mg * 4mg^–1^), and **(d)** protein content (% DW) among adult individuals of *N*. *inaequisculpta* collected four sites (MC2 to MC5) at progressive distance from a melting glacier (MC2 distal, MC5 proximal) Marian Cove, WAP. Different letters on box indicate significant differences among sites after Tukey’s HSD test. Measurements are given as average values ± SD (*n* = 80).

The values of lipid content per individual (mg * 4mg –1) showed statistically significant differences among sampling sites (one-way ANOVA, F_3,76_ = 12.30; *p* < 0.001). Individuals of the farthest site to the glacier (MC2: 0.70 ± 0.13 mg) presented higher amount of lipids than individuals from all other sampling sites (MC3: 0.52 ± 0.11 mg; MC4: 0.52 ± 0.11 mg; MC5: 0.49 ± 0.14 mg) (Fig 5A, S4 Table). Consistently, the percentage of lipids (% dry weight, DW) showed a tendency similar to that of total lipids (mg * 4mg^–1^) and significant differences were detected (one-way ANOVA, F_3,76_ = 12.30; *p* < 0.001). Individuals captured in MC2 had higher percentage of lipid (17.42 ± 3.24%) than individuals from the three others sites at different distances from the glacier (MC3: 12.92 ± 2.75%; MC4: 12.89 ± 2.77%; MC5: 12.16 ± 3.46%) (Fig 5B, S4 Table).

The protein content (mg * 4mg^–1^) showed significant differences among individuals of some study sites (Kruskal-Wallis test, H_3,80_ = 11.82; *p* < 0.01). Individuals of MC4 site presented a higher protein content (1.03 ± 0.20 mg) than individuals of MC5 site (0.84 ± 0.10 mg), whereas individuals of MC2 and MC3 sites presented similar values between them (MC2: 0.97 ± 0.25 mg; MC3: 0.93 ± 0.17 mg) (Fig 5C, S3 Table). Similarly, the percentage of proteins (%DW) were also statistically significant among individuals of some sampling sites (Kruskal-Wallis test, H_3,80_ = 11.82; *p* < 0.01) and displayed the same trend with highest protein percentages in the MC4 site (25.76 ± 4.88%) than individuals from the MC5 site (21.05 ± 2.46%). Individuals captured at the MC2 and MC3 sites showed similar values between them (MC2: 24.34 ± 6.12%, MC3: 23.22 ± 4.24%) (Fig 5D, S3 Table).

#### Energy content

**Fig 6:**
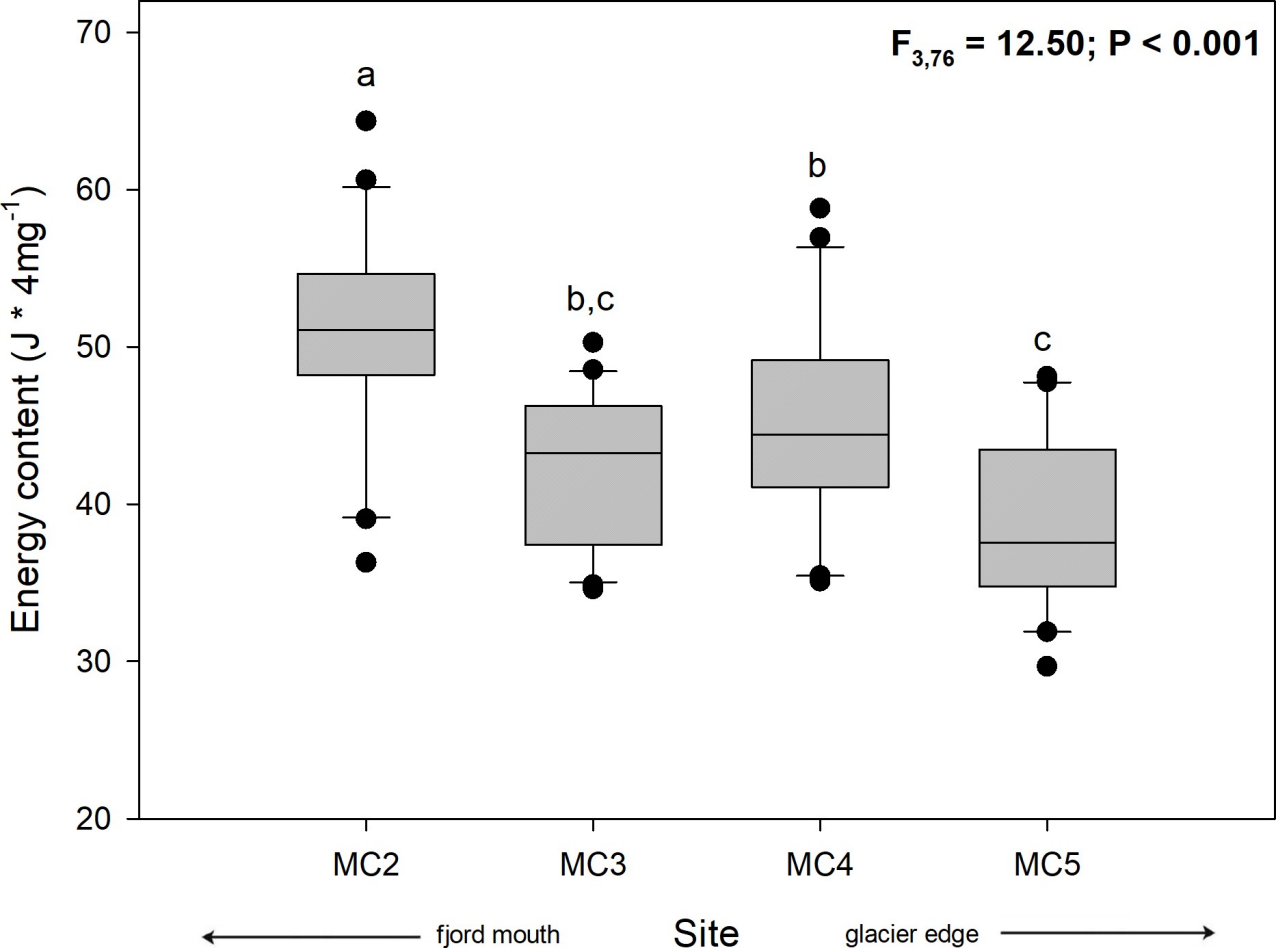
Boxplot of the spatial variation of energy content (J * 4mg^–1^) in adult individuals of *N*. *inaequisculpta* from four sites (MC2 to MC5) at progressive distance from a melting glacier (MC2 distal, MC5 proximal) in Marian Cove, WAP. Different letters on box indicate significant differences among sites after Tukey’s HSD test. Measurements are given as average values ± SD (*n* = 80).

Energy content per individual (J * 4mg^–1^) showed significant differences among sampling sites, where the individuals from the three sites farthest from the glacier showed a higher energy content (MC2: 50.57 ± 6.97 J; MC3: 42.39 ± 4.65 J; MC4: 44.74 ± 6.70 J) than individuals from the nearest site (MC5: 39.14 ± 5.80 J) (Fig 6, S4 Table). These differences were statistically significant among individuals from sites at different distances from the glacier (one-way ANOVA, F_3,76_ = 12.50; *p* < 0.001).

#### Fatty acids composition

The one-way ANOVA analyses, comparing each fatty acid among groups of individuals from the different sampling sites, did not detect significant differences (Table 1). For example, the proportion of total saturated fatty acids (SFA; MC2: 30.88 ± 3.21 mg FA * g dry weight, MC3: 33.54 ± 3.23 mg FA * g dry weight, MC4: 32.32 ± 3.16 mg FA * g dry weight, MC5: 30.80 ± 3.15 mg FA * g dry weight) (one-way ANOVA, F_3,11_ = 1.21; *p* = 0.32), and monounsaturated fatty acids (MUFA; MC2: 11.21 ± 2.60 mg FA * g dry weight, MC3: 12.20 ± 2.43 mg FA * g dry weight, MC4: 11.34 ± 2.51 mg FA * g dry weight, MC5: 11.30 ± 2.56 mg FA * g dry weight) (one-way ANOVA, F_3,11_ = 0.93; *p* = 0.44) was very similar across all study sites. Similarly, polyunsaturated fatty acids *n-6* (PUFA *n-6*; MC2: 3.00 ± 0.52 mg FA * g dry weight, MC3: 3.54 ± 0.14 mg FA * g dry weight, MC4: 3.39 ± 0.35 mg FA * g dry weight, MC5: 3.25 ± 0.43 mg FA * g dry weight) (one-way ANOVA, F_3,11_ = 2.14; *p* = 0.27), polyunsaturated fatty acids *n-3* (PUFA *n-3*; MC2: 5.25 ± 0.72 mg FA * g dry weight, MC3: 5.52 ± 0.57 mg FA * g dry weight, MC4: 5.47 ± 0.65 mg FA * g dry weight, MC5: 5.36 ± 0.69 mg FA * g dry weight) (one-way ANOVA, F_3,11_ = 3.03; *p* = 0.13), and total polyunsaturated fatty acids (total PUFA; MC2: 8.25 ± 0.59 mg FA * g dry weight, MC3: 9.06 ± 0.41 mg FA * g dry weight, MC4: 8.86 ± 0.50 mg FA * g dry weight, MC5: 8.61 ± 0.54 mg FA * g dry weight) (one-way ANOVA, F_3,11_ = 1.55; *p* = 0.36) also showed no significant differences among study sites (Table 1).

**Table 1:** Fatty Acid (FA) composition (expressed in mg FA * g dry weight^-1^ (DW) and in % of total FA pool in parentheses) of the soft tissue of *N*. *inaequisculpta* at different distances from a melting glacier in Marian Cove, WAP.

	Site			
Fatty acids	MC2	MC3	MC4	MC5
C11:0	1.34 ± 0.35 (2.66)	1.86 ± 0.80 (3.39)	1.48 ± 0.58 (2.82)	1.35 ± 0.40 (2.66)
C12:0	1.21 ± 0.21 (2.4)	1.40 ± 0.23 (2.55)	1.41 ± 0.27 (2.67)	1.41 ± 0.36 (2.78)
C14:0	2.18 ± 1.12 (4.33)	2.71 ± 0.97 (4.95)	2.66 ± 1.01 (5.06)	2.13 ± 0.85 (4.20)
C15:0	1.64 ± 0.55 (3.26)	1.59 ± 0.64 (2.90)	1.56 ± 0.57 (2.97)	1.37 ± 0.48 (2.70)
C16:0	10.86 ± 4.96 (21.57)	11.29 ± 5.05 (20.60)	10.94 ± 4.77 (20.83)	10.87 ± 4.84 (21.44)
C17:0	1.2 ± 0.58 (2.38)	1.49 ± 0.48 (2.72)	1.63 ± 0.71 (3.10)	1.47 ± 0.58 (2.90)
C18:0	6.89 ± 2.72 (13.69)	6.93 ± 2.87 (12.65)	6.87 ± 2.78 (13.08)	6.57 ± 2.79 (12.96)
C20:0	1.93 ± 0.84 (3.83)	1.95 ± 0.82 (3.56)	1.80 ± 0.83 (3.43)	1.93 ± 0.80 (3.81)
C22:0	1.68 ± 0.81 (3.34)	2.29 ± 0.86 (4.18)	2.01 ± 0.67 (3.83)	1.76 ± 0.77 (3.47)
C23:0	1.95 ± 0.83 (3.87)	2.03 ± 0.93 (3.70)	1.96 ± 1.00 (3.73)	1.94 ± 0.76 (3.83)
Total SFA	30.88 ± 3.21 (61.34)	33.54 ± 3.23 (61.20)	32.32 ± 3.16 (61.54)	30.80 ± 3.15 (60.74)
C14:1*n-5*	1.88 ± 1.00 (3.73)	2.54 ± 1.14 (4.64)	1.95 ± 0.78 (3.71)	1.88 ± 0.92 (3.71)
C16:1*n-9*	1.70 ± 1.06 (3.38)	1.73 ± 0.99 (3.16)	1.74 ± 1.06 (3.31)	1.80 ± 1.15 (3.55)
C18:1*n-9*	6.66 ± 2.63 (13.23)	6.61 ± 2.26 (12.06)	6.56 ± 2.32 (12.49)	6.62 ± 2.38 (13.05)
C22:1*n-9*	0.97 ± 0.46 (1.93)	1.32 ± 0.51 (2.41)	1.09 ± 0.42 (2.08)	1.00 ± 0.54 (1.97)
Total MUFA	11.21 ± 2.60 (22.27)	12.20 ± 2.43 (22.26)	11.34 ± 2.51 (21.59)	11.30 ± 2.56 (22.28)
C18:2*n-6*t	1.13 ± 0.46 (2.24)	1.67 ± 0.69 (3.05)	1.45 ± 0.67 (2.76)	1.32 ± 0.65 (2.60)
C20:4*n-6* (ARA)	1.87 ± 0.76 (3.71)	1.87 ± 0.85 (3.41)	1.94 ± 0.82 (3.69)	1.93 ± 0.70 (3.81)
Total n-6 PUFA	3.00 ± 0.52 (5.96)	3.54 ± 0.14 (6.46)	3.39 ± 0.35 (6.45)	3.25 ± 0.43 (6.41)
C20:3*n-3*	0.98 ± 0.48 (1.95)	1.24 ± 0.42 (2.26)	1.13 ± 0.48 (2.15)	1.02 ± 0.48 (2.01)
C20:5*n-3* (EPA)	2.40 ± 0.90 (4.77)	2.38 ± 0.85 (4.34)	2.42 ± 0.96 (4.61)	2.36 ± 0.78 (4.65)
C22:6*n-3* (DHA)	1.87 ± 0.75 (3.73)	1.90 ± 0.81 (3.47)	1.92 ± 0.89 (3.68)	1.98 ± 0.82 (3.90)
Total n*-3* PUFA	5.25 ± 0.72 (13.43)	5.52 ± 0.57 (10.07)	5.47 ± 0.65 (10.42)	5.36 ± 0.69 (10.57)
Total PUFA	8.25 ± 0.59 (16.39)	9.06 ± 0.41 (16.53)	8.86 ± 0.50 (16.87)	8.61± 0.54 (16.98)
Total FA	50.34 ± 2.60 (100)	54.80 ± 2.58 (100)	52.52 ± 2.55 (100)	50.71 ± 2.54 (100)
*n-3*/*n-6*	1.75 ± 0.59	1.56 ± 0.41	1.61 ± 0.50	1.65 ± 0.54
ARA/EPA	0.78 ± 0.37	0.79 ± 0.36	0.80 ± 0.34	0.82 ± 0.30
DHA/EPA	0.78 ± 0.37	0.80 ± 0.34	0.79 ± 0.35	0.84 ± 0.27

Mean ± SD, n = 80. Abbreviations are the following = SFA: saturated FA; MUFA: monounsaturated FA; PUFA: polyunsaturated FA; ARA: arachidonic acid; EPA: eicosapentaenoic acid; DHA: docosahexaenoic acid. SFA = sum of C11:0, C12:0, 14:0, 15:0, 16:0, 17:0, 18:0 C20:0, C22:0 and C23:0; MUFA = sum of 14:1*n*-5, 16:1*n*-9, 18:1*n*-9 and 22:1*n*-9; Total n*-6* PUFA = sum of 18:2*n*-6t and 20:4*n*-6; Total *n*-3 PUFA = sum of 20:3*n*-3, 20:5*n*-3, 22:5*n*-3 and 22:6*n*-3; Total PUFA = sum of *n*-3 and *n*-6 PUFA; Total FA = sum of Total SFA, Total MUFA, and Total PUFA.

PERMANOVA analysis which compared the overall fatty acid data, did not show significant differences among sampling sites (one-way PERMANOVA, Pseudo-F_3,76_ = 0.79; *p* = 0.61; 9999 permutations). The similarity among all groups is consistent with results from the SIMPER analysis, in which palmitic (C16:0), stearic (C18:0), myristic (C14:0), oleic (C18:1*n*-9), and EPA (C20:5*n*-3) fatty acids had the highest percentage of similarity contribution in all groups (S5 Table).

## Discussion

### Oceanographic parameters

Sea surface (top 10 m) parameters showed a lower salinity and chlorophyll-a concentration in sites farther away from the glacier edge, whereas temperature and dissolved oxygen decreased in sites nearest to the glacier (Fig 2). Water surface temperature followed a trend previously found by Yoo et al. [15] in the same study site, where it is clearly observed that nearby sites have a lower temperature than sites far away from the glacier. Strikingly, salinity does not show consistency with the pattern found in other surveys at the same study site [15], since sites near the glacier show a higher salinity (34.05 ± 0.03 PSU) than sites far from it (33.95 ± 0.008 PSU). It has been described that an adjacent site to the glacier is highly stratified unlike the farthest site from the glacier, recognizing four different water layers during summer [15]. The present study focused on the first water layer (top 10 m), which is characterized by cold and cloudy meltwater. In this context, although the data shown in this study are different, it may be due to a greater amount of non-melted ice at the nearest site to the glacier than at the farthest site to the glacier (personal observation). The lower amount of dissolved oxygen at the site near the glacier could be explained by an increased production of organic matter and/or bacterial respiration due to the increase in nutrients that are released during glacier melting [46].

The surface chlorophyll-a values recorded in this work (ca. 0.20–0.50 mg/m^3^) are within the ranges previously described for King George Island fjords. For example, a study made in Marian Cove between 1996–2008 recorded chlorophyll-a values between ca. 0.25–0.90 mg/m^3^ during November [47,48]. Consistently, another study made in Potter Cove between 1991–2009 recorded a range of chlorophyll-a values between ca. 0.20–1.30 mg/m^3^ during the same period [49]. The amount of chlorophyll-a found in this work can be influenced by a wide variety of factors. In the primary productivity context, iron is the most limiting micronutrient in the Southern Ocean [50] and its release in some areas of the WAP has been demonstrated due to glacial melting [51]. Similar results were found in Marian Cove, since increased release of important macro- and micronutrients (e.g. Fe and Mn) has been measured when the glacier melts into the seawater [52]. Therefore, although the dynamics in the Antarctic fjords are complex, this late spring nutrient release could play an important role in the primary productivity found in this work in the nearer sites to the melting glacier.

In bottom waters (bottom 10 m), significant differences were also found in the oceanographic parameters measured between the study sites. Lower temperature and chlorophyll-a, and a greater amount of dissolved oxygen and salinity, were found at sites nearer to the glacier compared to those farther (Fig 3). The temperature pattern reported here suggests that glacier melting could be lowering the temperature of the water adjacent to the glacier by releasing subglacial meltwater. The low levels of chlorophyll-a found in bottom waters, compared with chlorophyll-a found in surface waters, on the one hand could be a sign of high consumption of phytoplankton by primary consumers. On the other hand, perhaps only a portion of the organic matter exported to the benthic system in the last phytoplankton bloom event could be observed due to the influence of the bottom water currents. Salinity is within normal range of values described previously for Antarctic waters, while dissolved oxygen is over some values recently recorded at south of the WAP (i.e. mean salinity is around 34 PSU) [49], (dissolved oxygen between 6.57–7.65 mg/L approximately at 100 m depth) [51]. These values suggesting the differences found among sites may not have an effect on the biology/physiology of the studied species.

### Nutritional condition of *N*. *inaequisculpta*

Considering the technical difficulties for sampling in polar environments when the ocean is covered by sea-ice, the results presented here could be used as a first approach of spatial variation on the nutritional condition of this species during late spring. However, considering also that the nutritional status of individuals is likely to be affected by oceanographic parameters on a wide time scale, it is necessary to obtain samples at different seasons in futures studies (if possible) or use a mathematical model to predict how the nutritional condition of this species varies during an annual cycle. In this context, the oceanographic data (seawater temperature, salinity, dissolved oxygen, and chlorophyll-a) measured were within the natural environment ranges described previously for the WAP in different temporal scales [49,51,53,54]. Experiments performed on stenothermal Antarctic marine invertebrates that evaluate the effect of seawater temperature on the physiology (e.g. thermal tolerances), indicate that in general, there is a low capacity for acclimatization to the increased above natural environmental values [55,56,57]. However, the experimental factor values used in these studies are quite different than the values of the oceanographic parameters measured *in situ* in this work. For example, a study in Antarctic isopod species reported changes in some biological functions (e.g. reduction in the locomotor activity and weak reaction to food odour) when exposed to temperature increase (between 0 ºC and 5 ºC) and salinity decline (30–34 PSU) [58].

Additionally, the number of studies that assess the effect of oxygen concentration on Antarctic marine invertebrates has increased in the last decades [59]. In the context of climate change, in polar species it has been observed that large sized species are more sensitive to oxygen decrease than small sized species [60]. Although some species have followed this pattern (e.g. bivalve *Laternula elliptica*) [61,62], others did not support this hypothesis (e.g. 12 pycnogonid species) [63], suggesting divergent responses across species/taxa. In this context, although the previous hypothesis should not apply to our focal species due to its small size it is necessary to evaluate this topic in future studies. Nonetheless, our natural environment data showed dissolved oxygen values of 300 μmol/kg, which are similar to natural environment values reported previously for WAP at 100 m depth (160–300 μmol/kg) [51]. Thus, all the oceanographic data suggest that there were favorable environmental conditions for the physiological/biological performance of benthic marine fauna at Marian Cove at the time of sampling.

Regarding *N*. *inaequisculpta* nutritional condition/fitness results, and considering the slow tissue turnover in polar regions, the biochemical composition observed in the focal species could be reflecting the diet of a couple months ago [64]. In this context, it would be interesting to develop a year-round research to determine how the biochemical composition of this species changes in relation to the food available in the sediment. As for *N*. *inaequisculpta* biochemical composition, the present study shows that proteins are the main biochemical component of the species dry weight (21–25% DW), above the amount of lipids (12–17% DW). Comparatively, the lipid content values found in *N*. *inaequisculpta* are higher than those reported for some marine bivalves of temperate, sub-arctic and Antarctic regions (Table 2). Whereas, it is not possible to observe a clear pattern on the protein content of different marine bivalve species, which could be related to a large data variation and a lack of analytical methods standardization (Table 2). Furthermore, some results similar to ours on the biochemical composition in other Antarctic marine invertebrate species have been described [65]. For example, a higher protein content relative to lipid content was found in the marine Antarctic gastropods *Austrodoris kerguelensis*, *Tritoniella belli* and *Marseniopsis mollis* (protein: 7–25% DW, lipid: 6–18% DW) [66], in the ascidian *Cnemidocarpa verrucosa* (protein: 5.9–18% DW, lipid: 4.9–16.1% DW) [67] and in the nemertean *Parborlasia corrugatus* (protein: 17.9–36% DW, lipid: 7.9–13.8% DW) [65].

**Table 2:** Biochemical composition (lipid and protein, % dry weight) recorded in marine bivalve species of different regions.

Species	Region	Data period	Lipid (%DW)	Protein (%DW)	References
*N*. *inaequiscupta*	Antarctic	Late spring	12.2–17.1	21.1–25.8^a^	This study
*Laternula elliptica*	Antarctic	Summer	ca. 8.20	n/a	[68]
	Antarctic	Late spring	6.0–18.0	60.0–85.0^b^	[69]
*Aequiyoldia eightsii*	Antarctic	Summer	4.60–8.30	13.1–22.3^a^	Unpublished data
*Nucula sulcata*	Sub-arctic	Late spring	6.69–6.77	9.72–9.95^c^	[70]
*Nucula turgida*	Sub-arctic	Late spring	13.0–15.0	60.0–66.0^b^	[71]
*Abra alba*	Sub-arctic	Late spring	4.66–5.56	8.88–9.40^c^	[72]
*Chlamys septemradiata*	Sub-arctic	Late spring	5.89–13.1	9.51–13.8^c^	[73]
*Lima hians*	Sub-arctic	Late spring	5.31–7.36	8.10–10.5^c^	[74]
*Astarte montagui*	Sub-arctic	Late spring	4.12–5.58	9.35–9.97^c^	[75]
*Mytilus edulis*	Sub-arctic	Spring	5.00–6.00	50.0–60.0^b^	[76]
*Mytilus galloprovincialis*	Temperate	Late spring	1.35	11.1^b^	[77]
	Temperate	Summer	6.27	49.5^b^	[78]
	Temperate	Late spring	1.60	9.70^b^	[79]
	Temperate	Late spring	1.84	8.78^a^	[80]
*Tapes decussatus*	Temperate	Late spring	1.61	10.9^b^	[81]
*Tapes philippinarum*	Temperate	Late spring	1.33	10.5^b^	[81]
*Crassostrea gigas*	Temperate	Spring	7.33	ca. 16.0^a^	[82]
	Temperate	Spring	8.00	40.5^a^	[83]
*Ostrea edulis*	Temperate	Spring	6.90	38.9^a^	[83]
*Mactra chinensis*	Temperate	Spring	1.50–22.0	16.1–43.4^d^	[84]
*Fulvia mutica*	Temperate	Spring	ca. 7.50	ca. 55.0^b^	[85]

^a^ = protein data through Lowry method

^b^ = protein data through Kjeldhal method

^c^ = estimation of nitrogen content; n/a: not available.

In the case of *N*. *inaequisculpta*, the biochemical composition and energy content showed a peculiar result in the site MC3. Individuals of the MC3 site have a similar biochemical content to individuals of the other sites in 4 mg of dry weight, but when considering the total weight (ca. 10 mg), individuals of the site MC3 double their biochemical content relative to individuals from other sampling sites. This result could be related to an underwater sill found recently at the MC3 site using bathymetry multibeam equipment [86]. In biological terms, this underwater sill could be acting as a food retainer, allowing *N*. *inaequisculpta* individuals to invest a greater amount of energy in soft tissue growth due to a greater amount of available food.

The biochemical composition and energy content of *N*. *inaequisculpta* individuals within the fjord was higher in the farthest than in the nearest sites to the glacier (Figs 5 and 6), displaying an interesting spatial variation that to our knowledge has not previously been found in Antarctic marine bivalves. Concordantly, a recent study that evaluated the biochemical composition of the Antarctic polychaetes *Maldane sarsi antarctica* and *Notomastus latericeus*, also found higher amount of lipids and proteins at the farther sites to the melting glacier in coastal fjords [87]. Further, another study evaluating the impact of regional warming on the biochemical composition of the sediments (lipids, proteins and carbohydrates) found that sediments from sites with a recent ice-loss have a lower lipid and protein content and higher amount of carbohydrates compared to sites with an ancient ice loss [10]. By one hand, this could be evidence that new habitats formed by the retreat of glaciers (recently exposed sites) would be still unstable environments with low amounts of available energy that can only sustain a benthic community at early stages of the colonization process [12,88]. By the other hand, the presence of infaunal species could shed lights on the important role they play at new blue carbon sinks due to glaciers melting and retreat [89].

Fatty acids are lipid components that can provide high amounts of energy, compared to amino acids for example, by being oxidized, and are also part of membranes as well as other cellular structures and pathways [90]. Especially, polyunsaturated fatty acids (PUFA) fulfill vital functions in organisms, for instance, in the development of the nervous system [91], immune responses [92,93], growth [94] and as precursors of eicosanoids that are important for cellular processes [95]. Certain marine organisms do not have the ability to obtain these fatty acids *de novo*, so certain types of PUFAs, such as eicosapenaenoic acid (EPA; C20: 5*n-3*), DHA (C22: 6*n-3*) and arachidonic acid (ARA; C20: 4*n- 6*) are considered essential fatty acids because they can only be obtained through the intake of food [90]. However, recent studies indicate that some molluscs species, including bivalves, have the ability to synthesize some fatty acids due to the presence of specialized enzymes [96]. In this context, it would be important that future studies can answer this question, especially in the Antarctic ecosystem where these topics are not well understood.

Numerous studies have used fatty acids biomarkers in Antarctic species, mainly focusing on revealing the species’ trophic ecology [97,98,99,100]. According to the presence of certain fatty acids found in this study and using biomarkers of fatty acids available in the literature, we suggest that *N*. *inaequisculpta* have an omnivorous feeding behavior (Table 3). On the other hand, there is a lack of studies on fatty acids as nutritional condition proxies of Antarctic marine invertebrates. In the present work, *N*. *inaequisculpta* did not display significant differences in its proportions of total fatty acids (i.e. saturated, monounsaturated and polyunsaturated) among the studied sites along the fjord, which indicates a similar nutritional quality for the whole fjord. Then, the low amount of food available in the sediment could be the most likely cause of the observed pattern for the biochemical composition, as mentioned above.

**Table 3:** Fatty acid trophic markers in the benthic and pelagic environment.

Fatty acid biomarker	Food source	References
C15:0, C17:0	Bacteria	[101,102,103]
C16:0, C18:0, C20:0, C18:1*n-9*	Detritus	[96,102]
C18:1*n-9*, C18:2*n-6*, C20:4*n-6*, C20:5*n-3*	Brown algae	[96]
C18:2*n-6*	Green algae	[101,104]
C18:2*n-6*, C20:4*n-6*, C22:6*n-3*	Heterotrophic flagellates	[96]
C22:6*n-3*, C18:1*n-9*	Meiofauna	[96]
C22:1*n-9*	Zooplankton	[101]
C22:6*n-3*, C20:5*n-3*	Diatoms and dinoflagellates	[96,101,102,103,104]

The nutritional status of adults has a direct effect on reproduction, offspring success, stability of populations, and resilience of species in ecosystems [105]. Better nutritional condition of adults can be reflected in a higher investment of maternal energy in offspring and hence an immediate initial source of intraspecific variability [105], which will subsequently translate into cascading effects throughout a specimens’ life-cycle [106]. One aspect widely studied in marine invertebrates has been the successful development of gametes. In this context, it has been observed that individuals fed with diets of higher nutritional quality produce larger and better-quality gametes [107]. After fertilization, the produced larvae also had a better energetic quality, lower nutritional vulnerability, better ability to resist environmental changes, and therefore higher survival rates [108]. On the contrary, if parental individuals did not invest a sufficient amount of proteins, lipids, and fatty acids in their offspring, mortality was likely to increase, and malformations were more likely to occur during the development of the offspring, making them non-viable [105]. Therefore, individuals who are closer to melting glaciers, and who show a lower nutritional condition, could produce offspring with a higher nutritional vulnerability, reflected in decreased larval survival and population instability.

## Conclusions

Oceanographic data measured in this study showed spatial differences among study sites that could be related with the glacier melting. Also, oceanographic data were within the natural environment ranges described previously for the WAP for different temporal scales, suggesting no effect on the physiology or biology of *N*. *inaequisculpta* at the moment of sampling. On the other hand, individuals of this species display a spatial variation in the nutritional condition at different distances to the glacier. Specimens that lived in the nearest site to the glacier had a worse nutritional condition than individuals who lived in the farthest site to the glacier, likely related with the quantity not quality of food in sediments. Meanwhile, the higher fitness within the fjord, due to higher biomass of dry tissue, of this species is likely found at the intermediate site of the melting glacier transect. Thus, individuals who live near the glacier will likely have less reproductive success, less larval survival, and therefore a more unstable population. This could further lead to a decreased recycling capacity of nutrients by benthic species and, additionally, a negative effect on the carbon cycle in the WAP system.

## Supporting information

S1 Table Specification of the oceanographic sampling sites (vertical conductivity-temperature-depth profiles) and biological sampling sites (*N*. *inaequisculpta*), at different distances from a melting glacier in Marian Cove, WAP. (DOCX)

S2 Table Kruskal-wallis statistical summary for surface and depth oceanographic parameters of sites located at different distances from a melting glacier in Marian Cove, WAP. When significant differences were found, a multiple range test with a Bonferroni correction was used (*p < 0.05; **p < 0.01).
(DOCX)

S3 Table Statistical summary of the kruskal-wallis test of the nutritional parameters of the mollusc bivalve *N*. *inaequisculpta* from sites located at different distances from a melting glacier in Marian Cove, WAP. When significant differences were found, a multiple range test with a Bonferroni correction was used (***p < 0.001; **p < 0.01; ns in the superscript indicates that no significant differences were found).
(DOCX)

S4 Table Statistical summary of ANOVA for nutritional parameters of individuals of the bivalve mollusc *N*. *inaequisculpta* caught at different distances from a melting glacier in Marian Cove, WAP. Differences in all parameters [protein (mg * 4mg^–1^ and % DW), and energy content (J * 4mg^–1^)] were evaluated with a one-way ANOVA. When significant differences were found a Tukey HSD test was used; significant differences are indicated with asterisks (*p < 0.001).
(DOCX)

S5 Table Similarity percentage analysis (SIMPER) used to evaluate the contribution of each fatty acid found in individuals of *N*. *inaequisculpta* (*n* = 80) at different distances from a melting glacier in Marian Cove, WAP. (DOCX)

S1 File Nutritional condition data of the bivalve mollusc *N*. *inaequisculpta* (size, biomass, lipid, protein, fatty acids and energy content) in Marian Cove, WAP. (RAR)

S2 File Oceanographic variables data (seawater temperature, salinity, dissolved oxygen and chlorophyll) of top (top 10 m) and bottom (bottom 10 m) water layers in Marian Cove, WAP. (RAR)
