# Supplementary material for: The influence of glacial melt and retreat on the nutritional condition of the bivalve Nuculana inaequisculpta (Protobranchia: Nuculanidae) in the West Antarctic Peninsula
Source: PLoS One. 2020 May 21;15(5):e0233513. doi: 10.1371/journal.pone.0233513 (PMC7241748; doi:10.1371/journal.pone.0233513)
Supplement: S1 Table — (DOCX) [file pone.0233513.s001.docx]

**SI Table. Specification of the oceanographic sampling sites (vertical Conductivity-Temperature-Depth profiles) and biological sampling sites (*N. inaequisculpta*), at different distances from a melting glacier in Marian Cove, WAP.**

| Site | Coordinates | Distance from glacier (m) | Deployment date | Max deep (m) |
| --- | --- | --- | --- | --- |
| *Oceanographic* |  |  |  |  |
| MC 2 | 62°12'45.82''S; 58°46'8.04''W | 2695.87 | 11-24-2017 | 100 |
| MC 3 | 62°12'29.34''S; 58°44'52.33''W | 1489.43 | 11-24-2017 | 100 |
| MC 4 | 62°12'21.16''S; 58°44'23.49''W | 1002.43 | 11-24-2017 | 110 |
| MC 5 | 62°12'13.64''S; 58°43'53.32''W | 510.46 | 11-24-2017 | 70 |
| *Biological* |  |  |  |  |
| MC 2 | 62°12'45.72''S; 58°46'8.76''W | 2702.3 | 11-25-2017 | 102.9 |
| MC 3 | 62°12'29.16''S; 58°44'53.51''W | 1502.4 | 11-25-2017 | 102.7 |
| MC 4 | 62°12'20.52''S; 58°44'22.91''W | 986.3 | 11-25-2017 | 112.5 |
| MC 5 | 62°12'15.48''S; 58°44'3.84''W | 669.7 | 11-25-2017 | 112.8 |
