# Supplementary material for: The influence of glacial melt and retreat on the nutritional condition of the bivalve Nuculana inaequisculpta (Protobranchia: Nuculanidae) in the West Antarctic Peninsula
Source: PLoS One. 2020 May 21;15(5):e0233513. doi: 10.1371/journal.pone.0233513 (PMC7241748; doi:10.1371/journal.pone.0233513)
Supplement: S2 Table — When significant differences were found, a multiple range test with a Bonferroni correction was used (*p < 0.05; **p < 0.01). (DOCX) [file pone.0233513.s002.docx]

**S2 Table. Kruscal-Wallis statistical summary for surface and depth oceanographic parameters of sites located at different distances from a melting glacier in Marian Cove, WAP.**

| Parameter | Site | *n* | Ranks sum | H | *p* |
| --- | --- | --- | --- | --- | --- |
| *Surface* |  |  |  |  |  |
| Temperature (°C) | MC2 | 5 | 65 | 12.73 | < 0.01** |
|  | MC3 | 4 | 33 |  |  |
|  | MC4 | 3 | 16 |  |  |
|  | MC5 | 3 | 6 |  |  |
| Salinity (PSU) | MC2 | 5 | 30 | 8.53 | < 0.05* |
|  | MC3 | 4 | 19 |  |  |
|  | MC4 | 3 | 30 |  |  |
|  | MC5 | 3 | 41 |  |  |
| Dissolved oxygen (mg/L) | MC2 | 5 | 65 | 13.05 | < 0.01** |
|  | MC3 | 4 | 34 |  |  |
|  | MC4 | 3 | 15 |  |  |
|  | MC5 | 3 | 6 |  |  |
| Chlorophyll-a (mg/m^3^) | MC2 | 5 | 30 | 11.36 | < 0.01** |
|  | MC3 | 4 | 15 |  |  |
|  | MC4 | 3 | 33 |  |  |
|  | MC5 | 3 | 42 |  |  |
| *Depth* |  |  |  |  |  |
| Temperature (°C) | MC2 | 5 | 65 | 9.5 | < 0.01** |
|  | MC3 | 5 | 25 |  |  |
|  | MC4 | 5 | 30 |  |  |
| Salinity (PSU) | MC2 | 5 | 15 | 9.38 | < 0.01** |
|  | MC3 | 5 | 53 |  |  |
|  | MC4 | 5 | 52 |  |  |
| Dissolved oxygen (mg/L) | MC2 | 5 | 15 | 12.50 | < 0.01** |
|  | MC3 | 5 | 64 |  |  |
|  | MC4 | 5 | 40 |  |  |
| Chlorophyll-a (mg/m^3^) | MC2 | 5 | 65 | 9.5 | < 0.01** |
|  | MC3 | 5 | 30 |  |  |
|  | MC4 | 5 | 25 |  |  |

When significant differences were found, a multiple range test with a Bonferroni correction was used (**p* < 0.05; ***p* < 0.01).
