# Supplementary material for: The influence of glacial melt and retreat on the nutritional condition of the bivalve Nuculana inaequisculpta (Protobranchia: Nuculanidae) in the West Antarctic Peninsula
Source: PLoS One. 2020 May 21;15(5):e0233513. doi: 10.1371/journal.pone.0233513 (PMC7241748; doi:10.1371/journal.pone.0233513)
Supplement: S3 Table — When significant differences were found, a multiple range test with a Bonferroni correction was used (***p < 0.001; **p < 0.01; ns in the superscript indicates that no significant differences were found). (DOCX) [file pone.0233513.s003.docx]

| Parameter | Site | *n* | Ranks sum | H | *p* |  |
| --- | --- | --- | --- | --- | --- | --- |
| Shell length (mm) | MC2 | 40 | 2781 | 4.76 | 0.19^ns^ |  |
|  | MC3 | 40 | 3050 |  |  |  |
|  | MC4 | 40 | 3538 |  |  |  |
|  | MC5 | 40 | 3511 |  |  |  |
| Tissue dry weight (mg) | MC2 | 40 | 2216 | 63.52 | < 0.001*** |  |
|  | MC3 | 40 | 5198 |  |  |  |
|  | MC4 | 40 | 2581 |  |  |  |
|  | MC5 | 40 | 2884 |  |  |  |
| Protein (mg) | MC2 | 20 | 891 | 11.82 | < 0.01** |  |
|  | MC3 | 20 | 778 |  |  |  |
|  | MC4 | 20 | 1029 |  |  |  |
|  | MC5 | 20 | 541 |  |  |  |
| Protein (%DW) | MC2 | 20 | 891 | 11.82 | < 0.01** |  |
|  | MC3 | 20 | 778 |  |  |  |
|  | MC4 | 20 | 1029 |  |  |  |
|  | MC5 | 20 | 541 |  |  |  |

**S3 Table. Statistical summary of the Kruskal-Wallis test of the nutritional parameters of the mollusk bivalve *N. inaequisculpta* from sites located at different distances from a melting glacier in Marian Cove, WAP.**

When significant differences were found, a multiple range test with a Bonferroni correction was used (****p* < 0.001; ***p* < 0.01; ns in the superscript indicates that no significant differences were found).
