# Supplementary material for: The influence of glacial melt and retreat on the nutritional condition of the bivalve Nuculana inaequisculpta (Protobranchia: Nuculanidae) in the West Antarctic Peninsula
Source: PLoS One. 2020 May 21;15(5):e0233513. doi: 10.1371/journal.pone.0233513 (PMC7241748; doi:10.1371/journal.pone.0233513)
Supplement: S4 Table — Differences in all parameters [protein (mg * 4mg–1 and % DW), and energy content (J * 4mg–1)] were evaluated with a one-way ANOVA. When significant differences were found a Tukey HSD test was used; significant differences are indicated with asterisks (*p < 0.001). (DOCX) [file pone.0233513.s004.docx]

**S4 Table. Statistical summary of ANOVA for nutritional parameters of individuals of the bivalve mollusk *N. inaequisculpta* caught at different distances from a melting glacier in Marian Cove, WAP.**

| Parameter | Factor | df | MS | F | *p* |
| --- | --- | --- | --- | --- | --- |
| Lipid (mg) | Site | 3 | 0.19 | 12.30 | < 0.001* |
|  | Error | 76 | 0.02 |  |  |
|  | Total | 79 | 0.21 |  |  |
| Lipid (%DW) | Site | 3 | 115.98 | 12.30 | < 0.001* |
|  | Error | 76 | 9.43 |  |  |
|  | Total | 79 | 125.41 |  |  |
| Energy (J) | Site | 3 | 464.85 | 12.51 | < 0.001* |
|  | Error | 76 | 37.17 |  |  |
|  | Total | 79 | 502.02 |  |  |

Differences in all parameters [lipid (mg * 4mg^–1^ and % DW), and energy content (J * 4mg^–1^)] were evaluated with a one-way ANOVA. When significant differences were found a Tukey HSD test was used; significant differences are indicated with asterisks (**p* < 0.001).
