# Supplementary material for: The influence of glacial melt and retreat on the nutritional condition of the bivalve Nuculana inaequisculpta (Protobranchia: Nuculanidae) in the West Antarctic Peninsula
Source: PLoS One. 2020 May 21;15(5):e0233513. doi: 10.1371/journal.pone.0233513 (PMC7241748; doi:10.1371/journal.pone.0233513)
Supplement: S5 Table — (DOCX) [file pone.0233513.s005.docx]

| **Site** | **Similarity** | **FA*** | **Av.Abund** | **Av.Sim** | **Sim/SD** | **Contrib%** | **Cum.%** |
| --- | --- | --- | --- | --- | --- | --- | --- |
| MC2 | 94.26 | C16:0 | 1.79 | 7.4 | 18.49 | 7.85 | 7.85 |
|  |  | C18:0 | 1.6 | 6.66 | 14.74 | 7.07 | 14.92 |
|  |  | C18:1n9 | 1.59 | 6.62 | 19.41 | 7.02 | 21.94 |
|  |  | C20:5n3 | 1.23 | 5.12 | 10.49 | 5.44 | 27.38 |
|  |  | C23:0 | 1.17 | 4.86 | 18.36 | 5.16 | 32.54 |
|  |  | C14:0 | 1.19 | 4.86 | 12.97 | 5.16 | 37.69 |
|  |  | C22:6n3 | 1.16 | 4.85 | 22.04 | 5.15 | 42.84 |
| MC3 | 94.27 | C16:0 | 1.8 | 7.21 | 14.61 | 7.65 | 7.65 |
|  |  | C18:1n9 | 1.59 | 6.46 | 17.99 | 6.86 | 14.51 |
|  |  | C18:0 | 1.6 | 6.42 | 12.92 | 6.81 | 21.32 |
|  |  | C14:0 | 1.26 | 5.1 | 11.28 | 5.41 | 26.73 |
|  |  | C20:5n3 | 1.23 | 4.97 | 14.36 | 5.27 | 32 |
|  |  | C14:1n5 | 1.24 | 4.92 | 12.59 | 5.21 | 37.22 |
|  |  | C22:0 | 1.21 | 4.89 | 14.27 | 5.18 | 42.4 |
| MC4 | 94.01 | C16:0 | 1.79 | 7.31 | 18.02 | 7.77 | 7.77 |
|  |  | C18:0 | 1.6 | 6.53 | 12.88 | 6.95 | 14.72 |
|  |  | C18:1n9 | 1.58 | 6.53 | 17.54 | 6.94 | 21.66 |
|  |  | C14:0 | 1.26 | 5.13 | 11.83 | 5.46 | 27.12 |
|  |  | C20:5n3 | 1.23 | 5 | 13.85 | 5.32 | 32.44 |
|  |  | C22:0 | 1.18 | 4.85 | 20.14 | 5.16 | 37.6 |
|  |  | C20:4n6 | 1.16 | 4.78 | 15.91 | 5.08 | 42.69 |
|  |  | C14:1n5 | 1.16 | 4.73 | 13.6 | 5.03 | 47.72 |
|  |  | C23:0 | 1.16 | 4.7 | 12.53 | 5 | 52.72 |
| MC5 | 94.27 | C16:0 | 1.79 | 7.35 | 15.65 | 7.8 | 7.8 |
|  |  | C18:1n9 | 1.59 | 6.61 | 19.45 | 7.01 | 14.81 |
|  |  | C18:0 | 1.58 | 6.53 | 13.39 | 6.92 | 21.74 |
|  |  | C20:5n3 | 1.23 | 5.12 | 13.47 | 5.43 | 27.17 |
|  |  | C14:0 | 1.19 | 4.96 | 17.39 | 5.26 | 32.43 |
|  |  | C20:4n6 | 1.17 | 4.89 | 16.92 | 5.18 | 37.61 |
|  |  | C22:6n3 | 1.17 | 4.87 | 18.81 | 5.16 | 42.78 |

**S5 Table. Similarity percentage analysis (SIMPER) used to evaluate the contribution of each fatty acid found in individuals of *N. inaequisculpta* (*n* = 80) at different distances from a melting glacier in Marian Cove. WAP.**

The table shows fatty acids that contribute more than 5% to similarity; Av. Abund average = abundance of each fatty acid; Av. Sim = the average similarity that each fatty acid contributes; Sim / SD = the proportion of similarity and standard deviation; Contrib% = the contribution of each fatty acid to the general similarity; Cum.% = General additive similarity.
